# Non-Work-Related Use of Personal Mobile Phones by Hospital Registered Nurses

**DOI:** 10.2196/mhealth.4001

**Published:** 2015-01-13

**Authors:** Deborah L McBride, Sandra A LeVasseur, Dongmei Li

**Affiliations:** ^1^Samuel Merritt UniversityOakland, CAUnited States; ^2^Hawaii State Center for NursingUniversity of HawaiiManoa, HIUnited States; ^3^School of Medicine and DentistryUniversity of RochesterRochester, NYUnited States

**Keywords:** distraction, smartphone, cellular phone, Internet, nurses, hospital, non-work related smartphone use

## Abstract

**Background:**

Personal mobile phones and other personal communication devices (smartphones and tablet computers) provide users with an ever-increasing number and diversity of non-work-related activities while at work. In hospitals, where the vigilance of health care workers is essential for patient care, the potential distraction of these devices could be hazardous to patients.

**Objective:**

The objective of this study was to determine the frequency of non-work-related use of personal mobile phones and other personal communication devices among hospital registered nurses.

**Methods:**

In March 2014, a previously validated 30-question survey was emailed to the 10,978 members of the Academy of Medical Surgical Nurses. There were 825 respondents who met the inclusion criteria.

**Results:**

The use of a personal mobile phone or other personal communication device while working (excluding meal times and breaks) was reported by 78.1% (644/825) of respondents. Nurses reported regularly (sometimes, often, or always) sending personal emails and text messages (38.6%, 318/825), reading news (25.7%, 212/825), checking/posting on social networking sites (20.8%, 172/825), shopping (9.6%, 79/825), and playing games (6.5%, 54/825) while working.

**Conclusions:**

This study found that hospital nurses frequently use their personal mobile phones or other personal communication devices for non-work-related activities at work. The primary activity reported was to send personal emails and text messages to family and friends.

## Introduction

Personal mobile phones and other communication devices provide users with access to a wealth of electronic media such as the Internet, email, and texting, which help them fulfill tasks both at home and work. The work-related benefits of these devices to health care providers are numerous, including access to medical references, clinical tools, and patient information [1-6]. In addition to work-related sites, however, there is an ever-increasing number and diversity of recreational sites including games, gambling, and social networking. Previous research has reported that personal Internet use during working hours is increasingly common and that a majority of workers, regardless of age or occupational status, report using their personal mobile phones or other communication devices to engage in non-work-related activities in the workplace [7-13]. Personal mobile phones and other communication devices have the potential to distract health care providers from the vigilance required for patient care. Health care organizations are starting to take notice of this problem. The ECRI Institute (previously the Emergency Care Research Institute), a non-profit organization that uses applied scientific research to improve patient care, publishes an annual top 10 technology hazards list. “Caregiver distractions from smartphones and other mobile devices” was ninth on the list of health technology hazards for 2013 [14]. However, the extent of this issue in hospitals is unknown.

The objective of this study was therefore to determine the frequency of non-work-related use of personal mobile phones and other communication devices among hospital registered nurses.

## Methods

In March 2014, a recruitment email containing a link to a previously validated 30-question survey was sent to the 10,978 members of the Academy of Medical Surgical Nurses (AMSN) [15]. A total of 940 (8.56%) members completed the Web-based questionnaire and 825 (7.25%) met the inclusion criteria for the study of current full-time employment as a registered nurse in a hospital with an average of more than 5 hours a week of patient contact. The demographic distribution of the study sample of 825 was 48 (5.8%) men, 755 (93.9%) female; age ranges were 20-30 years (9.3%, 77/825), 31-40 years (18.1%, 141/825), 41-50 years (23.9%, 197/825), 51-60 years (39.2%, 323/), and >61 years (9.3%, 77/825).

The survey instrument was piloted in 2013 [15]. It consisted of four parts, with questions about (1) demographics, (2) the use of personal communication devices, (3) opinions about the effects of personal communication devices on the work of registered nurses, and (4) hospital policies concerning personal communication devices. The questions, which were developed based on a literature review and interviews with hospital nurses, asked respondents to rank the types of activities they engage in on a 5-point Likert scale to determine how frequently they participated in each activity. Psychometric testing of the questionnaire included examining internal consistency and test-retest reliability in a sample of 50 registered nurses. A Spearman rho correlation was used to determine the test-retest reliability. There was a strong test-retest reliability between the same test administered 1 week apart, with an average agreement for the Likert scale responses of 74% (range 43-100%). Accounting for responses within 1 SD range on the Likert scale increased the agreement to 96% (range 87-100%). The Cronbach coefficient alpha values examining the internal consistency in three of the domains were high: utilization (.84), impact (.96), and opinion (.85), with lower agreement in the performance domain (.45). Based on the results of the pilot survey, questions in the performance domain were rewritten to clarify the underlying concept of work performance.

## Results

### Overview

We examined the sample subsets to determine the representativeness of the sample relative to the United States nursing workforce data from The National Council of State Boards of Nursing and the Forum of State Nursing Workforce Centers 2013 National Workforce Survey for RNs [16]. The probability that the percentage of various subgroups in the study sample was representative of the larger population of the United States nursing workforce was calculated using a two-population *Z* test. The probabilities indicated that gender and location of primary place of employment (urban/rural) were represented appropriately in the study sample. Respondents in the age groups under age 40 years were underrepresented and age groups over age 55 years were overrepresented. The use of the AMSN membership list may have biased the age distribution of the survey sample toward older age groups. Whites, American Indians/Alaskan natives, and Native Hawaiian or Pacific Islanders were underrepresented, while Hispanic and multiple/other ethnicities were overrepresented. Consideration was given to weighting the study sample data for age and race/ethnicity, however several points argue against it. These include the small sample sizes within several age and race/ethnicity groups and the inherent subjectivity of racial/ethnic groups. While the response rate was low relative to other Web-based surveys, this may have been the result of the perceived sensitive nature of the subject, with respondents preferring not to admit that they had used their personal mobile phones and other communication devices at work for non-work related activities. In addition, Holbrook et al [17] assessed whether lower response rates were associated with decreases in the demographic representativeness of a sample. They examined the results of 81 national surveys with response rates varying from 5% to 54% and found that surveys with much lower response rates were only minimally less accurate. As a result of the issues described above, including the limitations associated with the study design and the available sample size, it was decided not to weight the current survey data, but to report the unweighted survey results with the recognition that the results, while valuable, may not be generalizable to the entire US registered nursing workforce.

### Primary Personal Communication Device

The majority of respondents (73.0%, 602/825) reported that their primary personal communication device was an enhanced mobile phone (mobile phone, texting, email, Internet access, and apps), 12.6% (104/825) a mobile phone with texting, 8.0% (66/825) a basic mobile phone, 2.8% (23/825) a tablet computer, and 1.3% (11/825) did not own a personal communication device.

### Frequency of Personal Mobile Phone or Other Communication Device Use

More than three-quarters (78.1%, 645/825) of respondents acknowledged that they always, often, or sometimes used their personal mobile phone or other communication device at work, excluding breaks or meal times (Table 1).

**Table 1:** Frequency of personal mobile phone or other communication device use while at work (n=825).

	Never	Rarely	Sometimes	Often	Always	No response
	n (%)					
How often do you use your personal mobile phone or other communication device while at work (excluding breaks and meal times)?	53 (6.4)	105 (12.7)	139 (16.8)	312 (37.8)	194 (23.5)	22 (2.7)

### Use of Personal Mobile Phone or Other Communication Device While at Work for Non-Work-Related Activities

Study participants were asked which non-work-related activities they used their personal mobile phone or other communication device for while working. These activities had previously been identified by researchers as potential uses of personal mobile phones at work [8,18-20].

Respondents reported using their personal mobile phone or other communication device always, often, or sometimes for calling or checking/sending personal emails or text messages (38.5%, 318/825), reading online news (25.7%, 212/825), checking/posting on social networking sites (20.8%, 172/), shopping (9.6%, 79/825), and playing games (6.5%, 54/825) (Table 2).

Non-work-related use of personal mobile phones or other communication devices at work was significantly correlated with age. Respondents under 30 years of age were more likely to use their personal mobile phone or other communication device at work for non-work-related activities than those over the age of 30 years. There was no correlation between personal mobile phone use and gender.

**Table 2:** Number of study respondents who answered the question, “On an average workday, describe your use of your personal mobile phone or other communication device (excluding breaks and meal times)?” (n=825).

	Never	Rarely	Sometimes	Often	Always	No response
	n (%)					
Read news	489 (59.3)	108 (13.1)	125 (15.2)	59 (7.2)	28 (3.4)	16 (1.9)
Call or check/send emails or text messages to family or friends	278 (33.7)	218 (26.4)	191 (23.2)	70 (8.5)	57 (6.9)	11 (1.3)
Shop	648 (78.5)	82 (9.9)	39 (4.7)	23 (2.8)	17 (2.1)	16 (1.9)
Check/post on social networking sites	565 (68.5)	129 (15.6)	129 (9.1)	21 (2.5)	22 (2.7)	13 (1.6)
Play games	692 (83.9)	65 (7.9)	33 (4)	11 (1.3)	10 (1.2)	14 (1.7)

## Discussion

### Principal Findings

The use of personal mobile phones and other communication devices is widespread in hospitals, with 78.1% (645/825) of registered nurses reporting using their personal mobile phone or other communication device while working. Only 6.4% (53/825) of respondents reported never using their personal mobile phone at work (Table 1). These results agree with earlier research that found high rates of personal communication device use by health care providers [17,20-22]. Calling or checking/sending emails and text messages to family and friends was the most commonly reported non-work-related activity. These results support Turkle’s theory of a “tethered self”, where humans use their personal communication devices to connect themselves constantly to other people and places, needing the continuing reassurance of developing and maintaining their group membership [23]. Other researchers have speculated about the emotional reassurance that comes from interacting with others though a mobile phone and how it helps alleviate the “fear of missing out”, a form of social anxiety that results from “a compulsive concern that one might miss out on an opportunity for social interaction, a novel experience, a profitable investment or other satisfying event” [24]. An alternative explanation for this use of mobile phones was reported by Lin et al [25], who studied the association between fatigue and Internet addiction in Taiwanese hospital nurses. They classified 6% to 10% of their study participants as Internet addicts, whose use of the Internet was associated with fatigue and a possible degradation of performance. They defined “nurse fatigue” as a subjective feeling of tiredness that persists despite periods of rest. It can be the result of several contributing factors, including high job demands, shift rotation work schedules, extended work shifts, and poor sleep quality. They speculated that accessing the Internet using mobile devices enabled registered nurses to recover from work-related fatigue. Coker [8] also speculated that use of mobile phones for workplace Internet leisure browsing allows workers to take short, unobtrusive breaks, enabling them to recover their concentration and restore their ability to focus. He found that use of mobile phones at work to access the Internet had a positive effect on productivity.

### Conclusions

Registered nurses in hospitals frequently use their personal mobile phones or other communication devices for non-work-related activities while working. Personal mobile phones allow nurses to meet their emotional needs by maintaining connections with family and friends while working. In hospitals, where vigilance is essential for patient care, the potential distraction of personal mobile phones could be hazardous to patients. However, non-work-related activities may have a positive effect on performance, allowing employees to restore their concentration, achieve a balance between work and personal life, reduce stress, and improve performance. Further study is needed answer the question of how personal mobile phones can be safely integrated into the work of hospital nurses.

## Abbreviations

AMSN: Academy of Medical Surgical Nurses

## References

[1] Dala-Ali BM, Lloyd MA, Al-Abed Y (2011). The uses of the iPhone for surgeons. Surgeon.

[2] Elias BL, Fogger SA, McGuinness TM, D'Alessandro KR (2014). Mobile apps for psychiatric nurses. J Psychosoc Nurs Ment Health Serv.

[3] Franko OI (2011). Smartphone apps for orthopaedic surgeons. Clin Orthop Relat Res.

[4] Jensen Ang WJ, Hopkins ME, Partridge R, Hennessey I, Brennan PM, Fouyas I, Hughes MA (2014). Validating the use of smartphone-based accelerometers for performance assessment in a simulated neurosurgical task. Neurosurgery.

[5] Sohn W, Shreim S, Yoon R, Huynh VB, Dash A, Clayman R, Lee HJ (2013). Endockscope: using mobile technology to create global point of service endoscopy. J Endourol.

[6] Zuo KJ, Guo D, Rao J (2013). Mobile teledermatology: a promising future in clinical practice. J Cutan Med Surg.

[7] Black E, Light J, Paradise Black N, Thompson L (2013). Online social network use by health care providers in a high traffic patient care environment. J Med Internet Res.

[8] Coker BLS (2011). Freedom to surf: the positive effects of workplace Internet leisure browsing. New Technology, Work and Employment.

[9] Lim VK, Chen DJ (2012). Cyberloafing at the workplace: gain or drain on work?. Behaviour & Information Technology.

[10] Mastrangelo PM, Everton W, Jolton JA (2006). Personal use of work computers: distraction versus destruction. Cyberpsychol Behav.

[11] Prasad S, Lim VK, Chen DJ (2010). PACIS Proceedings, Paper 159.

[12] Vitak J, Crouse J, LaRose R (2011). Personal Internet use at work: Understanding cyberslacking. Computers in Human Behavior.

[13] Ozdalga E, Ozdalga A, Ahuja N (2012). The smartphone in medicine: a review of current and potential use among physicians and students. J Med Internet Res.

[14] ECRI (2012). Top 10 health technology hazards for 2013. Health Devices.

[15] McBride DL, Levasseur SA, Li D (2013). Development and validation of a web-based survey on the use of personal communication devices by hospital registered nurses: pilot study. JMIR Res Protoc.

[16] Budden JS, Zhong EH, Moulton P, Cimiotti JP (2013). Supplement: The National Council of State Boards of Nursing and the Forum of State Nursing Workforce Centers 2013 National Workforce Survey of Registered Nurses. J Nurs Regul.

[17] Holbrook A, Krosnick J Pfent A, Lepkowski JM, Tucker NC, Brick M, DeLeeuw ED, Japec L, Lavrakas PJ (2008). The causes and consequences of response rates in surveys by the news media and government contractor survey research firms. Advances in Telephone Survey Methodology.

[18] Koehler N, Vujovic O, McMenamin C (2013). Healthcare professionals’ use of mobile phones and the internet in clinical practice. JournalMTM.

[19] Lim VKG (2002). The IT way of loafing on the job: cyberloafing, neutralizing and organizational justice. J Organiz Behav.

[20] Smith T, Darling E, Searles B (2011). 2010 Survey on cell phone use while performing cardiopulmonary bypass. Perfusion.

[21] Katz-Sidlow RJ, Ludwig A, Miller S, Sidlow R (2012). Smartphone use during inpatient attending rounds: prevalence, patterns and potential for distraction. J Hosp Med.

[22] Wu R, Rossos P, Quan S, Reeves S, Lo V, Wong B, Cheung M, Morra D (2011). An evaluation of the use of smartphones to communicate between clinicians: a mixed-methods study. J Med Internet Res.

[23] Turkle S, Katz J (2008). Always-on/always-on-you: the tethered self. Handbook of Mobile Communication Studies.

[24] Przybylski AK, Murayama K, DeHaan CR, Gladwell V (2013). Motivational, emotional, and behavioral correlates of fear of missing out. Computers in Human Behavior.

[25] Lin SC, Tsai KW, Chen MW, Koo M (2013). Association between fatigue and Internet addiction in female hospital nurses. J Adv Nurs.

